# Fear and anger have opposite effects on risk seeking in the gain frame

**DOI:** 10.3389/fpsyg.2015.00253

**Published:** 2015-03-10

**Authors:** Marianne Habib, Mathieu Cassotti, Sylvain Moutier, Olivier Houdé, Grégoire Borst

**Affiliations:** ^1^Paragraph Laboratory, Department of Psychology, Paris 8 University, Paris Lumières University, Paris, France; ^2^CNRS Unit 8240, Laboratory for the Psychology of Child Development and Education, Department of Psychology, Paris Descartes University - Sorbonne Paris Cité, and Caen University, Paris, France; ^3^Institut Universitaire de France, Paris, France; ^4^Laboratoire de Psychopathologie et Processus de Santé, Department of Psychology, Paris Descartes University, Paris, France

**Keywords:** emotion, decision making, framing effect, anger, fear

## Abstract

Emotions strongly influence our decisions, particularly those made under risk. A classic example of the effect of emotion on decision making under risk is the “framing effect,” which involves predictable shifts in preferences when the same problem is formulated in different ways. According to dual process theories, this bias could stem from an affective heuristic belonging to an intuitive type of reasoning. In this study, we examined whether specific incidental negative emotions (i.e., fear and anger) influence framing susceptibility and risk-taking identically. In each trial, participants received an initial amount of money, and pictures of angry or fearful faces were presented to them. Finally, participants chose between a sure option and a gamble option of equally expected value in a gain or loss frame. Risk-taking was modulated by emotional context: fear and anger influenced risk-taking specifically in the gain frame and had opposite effects. Fear increased risk-averse choices, whereas anger decreased risk-averse choices, leading to a suppression of the framing effect. These results confirm that emotions play a key role in framing susceptibility.

## INTRODUCTION

Decision making under risk is based on an appraisal of different options offering various probabilities of winning and outcome values. Intuition and emotions appear to play important roles in this process, sometimes leading to decisional errors (Lerner and Keltner, 2000, 2001; Houdé and Tzourio-Mazoyer, 2003; Evans, 2008; Blanchette and Richards, 2010; Cassotti et al., 2012; De Neys and Bonnefon, 2013). A well-known decisional bias is the violation of the description invariance principle (Tversky and Kahneman, 1981; Kahneman and Frederick, 2007). According to this principle, preferences among prospects should not be affected by variations in the irrelevant features of the options, such as how they are described. However, converging evidence demonstrates predictable shifts in preferences when a given problem is framed in different ways, i.e., the “framing effect” (Tversky and Kahneman, 1981; De Martino et al., 2006; Cassotti et al., 2012). Classically, a framing effect occurs when participants make more risk-seeking choices when the outcome is formulated in terms of losses than when it is formulated in terms of gains (Tversky and Kahneman, 1981).

Many authors have postulated that decisional biases arise from a competition between two distinct types of reasoning, i.e., an intuitive-heuristic form of reasoning—Type 1, and an executive-analytic form of mental operations—Type 2 (Sloman, 1996; De Neys, 2006; Evans, 2011; Kahneman, 2012). The Type 1 processing operates quickly, has a high capacity and is independent of working memory and cognitive ability. On the other hand, the Type 2 processing is relatively slow, has a lower capacity and is heavily dependent on working memory and related to individual differences in cognitive ability (Evans, 2011). In some daily situation, a competition can arise between both types of reasoning and the relying on the Type 1 can conduct to decisional biases. According to Kahneman and Frederick (2007), the framing effect occurs because of an affective heuristic that belongs to Type 1 processing and conducts to a shift of preferences according to the formulation of the options (intuitive-heuristic behavior), thereby violating the invariance principle (analytic behavior). This affective heuristic arises from a strong attractiveness of the sure gains on the one hand, and a high aversion of the sure losses on the other hand. Recent neuroimaging and behavioral studies have provided evidence in support of this assumption (De Martino et al., 2006; Cheung and Mikels, 2011; Cassotti et al., 2012). In a study by De Martino et al. (2006), in each trial, the participants were given an initial amount of money (e.g., 50£) and were confronted with a choice between a sure outcome and a gamble of equally expected value, represented as a “wheel of fortune.” The sure prospect was framed in one of two ways: in the gain frame, the participants could “keep” a part of the initial amount (e.g., keep 20£), and in the loss frame, the participants could “lose” a part of the initial amount (e.g., lose 30£). Thus, the expected values were identical for the sure and gamble options in both frames. Greater activation of the amygdala, a brain region reported to play an important role in the processing of emotional stimuli (see for example Van Den Bulk et al., 2014), was reported when the participants demonstrated a typical framing effect. Thus, the participant’s tendency to be susceptible to the frame seems to be significantly related to emotional processes, supporting the hypothesis that the framing effect is driven by an affective heuristic (Type 1—De Martino et al., 2006). Conversely, the participants’ ability to control this bias and run counter to the framing effect (Type 2)—operationalized by a “rationality index”—was related to the degree of orbitofrontal cortex, medial prefrontal cortex and anterior cingulate cortex activation. These results suggest that the ability to resist to the framing effect is based on the detection of a conflict between a heuristic choice and an analytic choice and on the inhibition of the impulsive response. Meanwhile, the orbito-medial prefrontal cortex enables to integrate emotional and cognitive information (as the expected value of each choice) which in turns lead to a more rational behavior. Thus, the framing effect occurs when intuitive-emotional reactions interfere with one’s ability to reason according to the invariance principle (De Martino et al., 2006; Kahneman and Frederick, 2007).

Behavioral studies exploring the influence of emotional regulation and incidental emotions on framing susceptibility have also provided converging evidence that the framing effect could stem from an affective heuristic belonging to an intuitive type of reasoning. In a framing task adapted from De Martino et al. (2006), Cheung and Mikels (2011) demonstrated that risk-taking decreased significantly compared to a standard control condition when the participants were asked to “not let their emotions influence their choices” (emotion-regulation condition). In addition, risk-taking was related to the extent to which the participants stated that they relied on their emotions when making their choices. In a second experiment, the participants rated how they felt about their decision (i.e., from very negative to very positive). Positive affect increased risk taking in the loss frame, but not in the gain frame, whereas negative affect had no effect (Cheung and Mikels, 2011). Finally, one study directly investigated the effects of positive and negative incidental emotions on the framing effect (Cassotti et al., 2012). Incidental emotions, in contrast to integral emotions, refer to emotions that arise from task-irrelevant factors such as participants’ emotional states (Blanchette and Richards, 2010; Bandyopadhyay et al., 2013). In this study, the participants performed a classical framing task adapted from De Martino et al. (2006), in which pleasant or unpleasant pictures were presented before each choice. The incidental positive emotional context reduced risk-taking in the loss frame and led to a suppression of the framing effect. Thus, the positive context seems to reduce the affective impact of a sure loss and consequently reduce loss aversion. Consistent with Cheung and Mikels (2011), the incidental negative context did not influence framing susceptibility. The opposite impact of positive emotions on risk taking in the loss frame could be attributed to methodological differences in the evaluation and the impact of positive emotions. Cheung and Mikels (2011) evaluated affective ratings about participant’s choice, whereas Cassotti et al. (2012) manipulated an incidental emotional context—i.e., irrelevant for the task at hand—by presenting pictures with a positive emotional content before every trial.

Together, these results suggest that relying on affects, particularly positive affect, influences risk-taking in the framing task, and reinforce the view that the framing effect arises from an affective heuristic. However, the absence of an effect of negative emotions on the framing effect could initially appear to be surprising because of the many studies that have emphasized the significant effect of negative emotions on decision making (e.g., Lerner and Keltner, 2001; Blanchette and Richards, 2010). For instance, the neural circuitry of fear and anxiety (i.e., the amygdala and ventromedial prefrontal cortex) strongly overlaps with the neural circuitry involved in decision making and framing susceptibility (Hartley and Phelps, 2012). The Appraisal Tendency Framework (ATF; Lerner and Keltner, 2000, 2001) could provide a possible explanation for the absence of an effect of the negative emotional context on framing susceptibility (Cheung and Mikels, 2011; Cassotti et al., 2012). According to the ATF, specific emotions can differently affect judgment and risk-taking tendencies in function of their appraisal patterns. For example, fear and anger are two basic emotions with negative valence, but fear is associated with a sense of uncertainty and a tendency to perceive situational control in new situations, while anger is associated with a sense of certainty and individual control (see Smith and Ellsworth, 1985; Kuppens et al., 2003; for comparative influence of fear and anger). Therefore, fearful people should perceive greater risk across new situations. This perception will push them to be more risk-averse. Angry people should perceive less risk in new situations, their optimistic risk assessment should lead them to be more risk-seeking (Lerner and Keltner, 2000). Previous studies that investigated the effects of negative emotions on framing susceptibility did not account for the distinct and opposite influences of these two negative emotions. Cassotti et al. (2012) and Cheung and Mikels (2011) suggested that additional studies would be necessary to determine the effect of specific negative emotions on the framing effect.

Thus, if we want to fully understand the effects of emotions on decision making, we have to go beyond mere valence and investigate the effect of specific emotions. Investigating the specific influence of negative emotions would provide crucial information to better understand how negative emotions can influence risk-seeking behaviors in the framing effect. Using questionnaires assessing dispositional fear and anger, state affect and risk perception, Lerner and Keltner (2000) have demonstrated that fearful and angry individuals tend to assess differently the level of risk of their environment. Fear predicted higher risk assessments and fearful individuals expressed a preference for the sure option in the Asian disease problem (Tversky and Kahneman, 1981). In contrast, angry individuals perceived lower risk and chose predominantly the risky option (Lerner and Keltner, 2000, 2001). However, the transitory effects of fear and anger—produced by an emotional context—on risk-taking have not yet been studied.

The present study investigated whether specific incidental emotions (i.e., fear and anger) differently influenced framing susceptibility in risky choices and risk-taking in a monetary framing task. The participants were presented with either a picture of a fearful or angry face before choosing between a sure option (keeping or losing a given amount of money, in the gain and the loss frames, respectively) or a risky option (i.e., gamble the entire amount of money). Consistent with the ATF, we assumed that fear and anger would influence risk-taking in opposite ways. Incidental fear should decrease risk-taking (i.e., more sure option choices), whereas incidental anger should increase risk-taking (i.e., more risky option choices). In the control condition (no face displayed), we expected the participants to show a classical framing effect.

## MATERIALS AND METHODS

### PARTICIPANTS

Sixty-seven undergraduate university students (*M* = 21.75 years, SD = 1.90, 32 men) volunteered to participate in this study. All participants were naive regarding the experimental aims and were randomly assigned to one of the three experimental conditions. They were not monetarily rewarded in exchange for their participation. Participants were tested in accordance with national and international norms governing the use of human research participants and gave their informed consent before participating to the study.

### PROCEDURE

**FIGURE 1:**
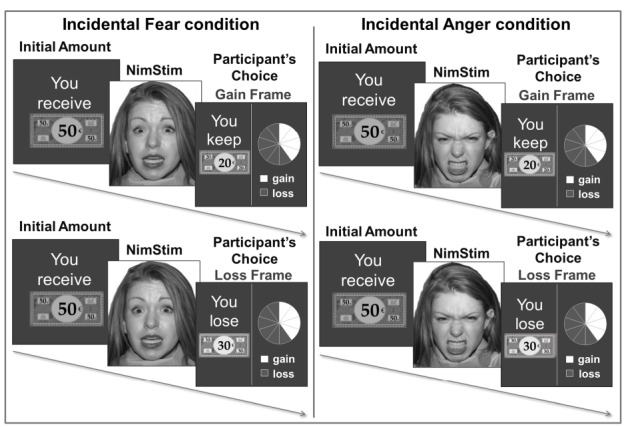
**Examples of trials presented in the gain and loss frames with an incidental emotional context of fear or anger.** In each trial, the participants received an initial amount of money depicted by a monopoly bill (e.g., 50€). A picture from the NimStim Face Stimulus Set (Tottenham et al., 2009) was then presented: a fearful face in the incidental fear condition and an angry face in the incidental anger condition. Finally, the participants chose between a sure and a gamble option of equally expected value in a gain frame or in a loss frame.

The participants completed a computerized gambling task adapted from Cassotti et al. (2012). The experiment employed three conditions: an incidental fear condition, an incidental anger condition and a control condition. In the fear and anger conditions, each choice was preceded by the presentation of a fearful or an angry face. In the control condition, no face was presented. During the practice session, the participants were familiarized with the gambling task and provided two practice trials. During the test session, the participants performed 70 trials: 25 trials framed in terms of gain, 25 trials framed in terms of loss and 20 catch trials. In each trial, they were provided with an initial amount of money for 2,500 ms (e.g., 50€) and then asked to choose between a sure option and a gamble option (see Figure 1). The gamble option was a wheel of fortune, depicting the probability of winning or losing the entire initial amount. The sure option could be formulated differently according to the frame. In the gain frame, the participants could “keep” a part of the initial amount (e.g., keep 20€), and in the loss frame, the participants could “lose” a part of the initial amount (e.g., lose 30€).

For the 50 test trials, expected values were identical for the sure and gamble options, and the framing conditions were mathematically equivalent. The initial amount varied between 10 and 50€ in increments of 10€. For each amount of money and frame, the probabilities of winning in the gamble options ranged from 30 to 70% in increments of 10%. The 20 catch trials were trials with noticeably different expected values between the sure and gamble options. In one-half of these trials, the gamble option was highly preferable, and for the other one-half of the trials, the sure option was preferable (e.g., a 10% probability of winning by choosing the gamble option versus a sure choice of 50% of the initial amount). The catch trials were designed to ensure that the participants were actively engaged in the task. The participants who obtained a percentage of success lower than 85% on the catch trials were excluded from the final sample (see Talmi et al., 2010). Two participants from the control condition and one from the incidental fear condition were excluded from the study. Thus, the final sample was composed of 22 participants in the incidental anger condition, 20 participants in the incidental fear condition, and 21 participants in the control group without emotional context.

In the incidental fear and the incidental anger conditions, pictures of faces were displayed for 3,000 ms after the presentation of the initial amount, with a fearful face in the incidental fear condition and an angry face in the incidental anger condition (see Figure 1). We selected 35 pictures of faces with a fearful expression and 35 with an angry expression (17 men and 18 women) from the NimStim Face Stimulus Set (Tottenham et al., 2009). We selected pictures of faces for which the emotions were correctly identified by over 70% of the participants.

### MANIPULATION CHECK

To determine whether the presentation of emotional faces could create incidental emotional contexts in the framing task (i.e., fear and anger contexts), we conducted a control study on 26 participants (mean age = 21.67 ± 1.09, 11 men). Participants were instructed to look at the pictures of faces presented on a computer screen. As in the framing task, each picture was displayed for 3,000 ms. Participants either saw the 35 fearful faces or the 35 angry faces presented in the framing task. Before and after presentation of the pictures, the participants were asked to rate on a 10-point scale to what extent each of 17 mood adjectives characterized their current emotional state (adapted from the Brief Mood Behavioral scale, see Mayer and Gaschke, 1988). We restricted our analyses to the ratings on the “fearful” and “angry” items. A 2 (facial expression: fearful vs. angry) × 2 (mood adjectives: fearful vs. angry) × 2 (conditions: pre-test vs. post-test) analysis of variance (ANOVA) on the ratings revealed a significant three-way interaction between facial expression, mood adjectives and condition, *F*(1,22) = 9.27, *p* < 0.01, ${\text{η}}_{\text{p}}^{\text{2}}$ = 0.30: participants reported being more fearful after the presentation of fearful faces (*M* = 1.42 ± 1.08 in the pre-test and *M* = 3.5 ± 2.07 in the post-test), *t*(11) = 3.57, *p* < 0.005, *d* = 1.26, but not more angry (*M* = 1.17, ± 1.11 in the pre-test and *M* = 1.75 ± 1.96 in the post-test), *t*(11) = 1.17 *p* = 0.53. In contrast, participants reported being more angry after the presentation of angry faces (*M* = 2 ± 2 in the pre-test and *M* = 3 ± 2.09 in the post-test), *t*(11) = 2.71, *p* < 0.05, *d* = 0.49, but not more fearful (*M* = 1.25 ± 1.49 in the pre-test and *M* = 1.25 ± 1.71 in the post-test), *t* < 1 (all p-values were corrected with a Bonferroni procedure). Thus, we are confident that, in the framing task, the presentation of the fearful faces created an incidental fearful context and that the presentation of the angry faces created an incidental anger context.

## RESULTS

To evaluate the effect of the incidental emotional context on risk-seeking in both frames, we conducted a 3 (conditions: incidental fear vs. incidental anger vs. control; between-participants factor) × 2 (frames: gain vs. loss; within-participants factor) × 5 (magnitude of outcomes: 10, 20, 30, 40, 50) mixed-design ANOVA. This analysis revealed a typical framing effect; the participants more frequently chose the gamble option in the loss frame (*M* = 54.9 ± 17.2%) compared to the gain frame (*M* = 36.8 ± 21.7%) when the three conditions were considered together, *F*(1,61) = 64.07, *p* < 0.0001, ${\text{η}}_{\text{p}}^{\text{2}}$ = 0.51. The main effect of condition was not significant, *F*(2,61) = 2.87, *p* = 0.064. Notably, the participants chose the gamble option in the gain and loss frames to different extents in the three conditions, as reflected by the significant interaction between condition and frame, *F*(2,61) = 9.84, *p* < 0.001, ${\text{η}}_{\text{p}}^{\text{2}}$ = 0.24. However, the mixed-design ANOVA did not reveal any significant interaction between condition and magnitude of outcomes, *F* = 1.46, *p* = 0.17, nor between condition, frame and magnitude of outcomes, *F* = 1.59, *p* = 0.13.

**FIGURE 2:**
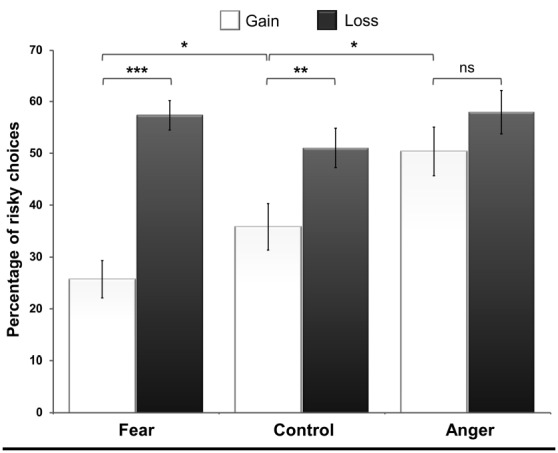
The percentage of risky choices in the incidental fear condition, control condition and incidental anger condition in the gain and the loss frames, *p < 0.05, **p = 0.005, ***p < 0.001, ns = not significant.

In the gain frame, the planned comparisons revealed that the participants more frequently chose the gamble option in the incidental anger condition than in the control condition (*M* = 50.4 ± 23.8% and *M* = 35.8 ± 19.5%, respectively), *t*(61) = 2.07, *p* < 0.05, *d* = 0.67. The participants also more frequently chose the gamble option in the gain frame in the control than in the incidental fear condition (*M* = 35.8 ± 19.5% and *M* = 24.8 ± 14.2%, respectively), *t*(61) = 1.78, *p* < 0.05, *d* = 0.64 (see Figure 2). *Post hoc* comparisons using Tukey’s Honestly Significant Difference tests revealed that the participants more frequently chose the gamble option in the loss frame (*M* = 57.6 ± 11.6%) than in the gain frame (*M* = 24.8 ± 14.2%) in the incidental fear condition, *p* < 0.001, *d* = 2.5. Similarly, in the control condition, the proportion of the chosen gamble option was higher in the loss frame (*M* = 51 ± 16.5%) than the gain frame (*M* = 35.8 ± 19.5%), *p* < 0.01, *d* = 0.84. In contrast to the two other conditions, we observed no framing effect in the incidental anger condition, (*M* = 58 ± 21.4% in the loss frame and *M* = 50.4 ± 23.8% in the gain frame), *p* = 0.32, *d* = 0.34. Risk-seeking did not differ across the three conditions in the loss frame, all *p*s > 0.10.

## DISCUSSION

The purpose of the present study was to investigate the influence of specific incidental negative emotions (i.e., fear and anger) on framing susceptibility and risk-seeking behaviors. Therefore, before they made their decision, the participants were presented with pictures of faces with either fearful or angry expressions. Critically, fear and anger have opposite effects on risk-taking in the gain frame, which in turn modulates the amplitude of the framing effect.

First, the participants in the control condition gambled more frequently in the loss frame than in the gain frame, a typical framing effect, which replicates previous findings (e.g., Tversky and Kahneman, 1981; De Martino et al., 2006; Cassotti et al., 2012). Second, in the incidental fear condition, we observed an increase of risk-averse choices in the gain frame compared to the control condition. Due to this increase, the framing effect was larger in the incidental fear condition compared to the control condition. Third, in the incidental anger condition, the decrease of risk-averse choices in the gain frame led to a suppression of the framing effect; after having seen angry faces, the participants were no longer affected by the formulation of the options, i.e., the proportion of risky choices did not differ across the frames. In this condition, the absence of a framing effect was due to an increase in risk-taking in the gain frame. This result further suggests that an incidental context of anger can significantly contribute to increased risk-taking, as predicted by the ATF (Lerner and Keltner, 2000, 2001). Thus, the effects of incidental negative emotions reported in this study confirm previous results demonstrating that fear and anger affect risk-aversion in opposite ways (Lerner and Keltner, 2000, 2001; Kugler et al., 2012).

According to the ATF (Lerner and Keltner, 2000), fear is defined by a better perception of uncertainty and situational control, whereas anger is defined by a better perception of certainty and individual control. As certainty and control are two factors that greatly influence risk perception and risk taking, fearful and angry people should demonstrate opposite patterns of risk taking. The results obtained in this study extended this question by investigated the transient effect of incidental fear and anger. In the current study, participants in the incidental fear condition are less prone to risk taking, whereas participants in the incidental anger condition demonstrate an increase of risky choices in the gain frame compared to participants in the control condition. According to the ATF, these differences in risk taking patterns confirm that incidental fear contributes to an increase of risk perception whereas incidental anger reduces risk perception, which leads to an increase of gambling choices. However, these results are restricted to the gain frame.

The incidental fear and anger conditions did not influence risk taking in the loss frame compared to the control condition. These results are in contradiction with the ones obtained by Lerner and Keltner (2001) and cannot be fully explained by the ATF framework. However, their work has examined the impact of trait-fear and trait-anger on risk-taking whereas the present study investigated the impact of the emotional context in which the decision is occurring. One possible explanation could be that anger and fear have differential impacts on risk perception, but only when the intuitive heuristic does not already exert a strong influence on risk perception. In the loss frame, the increase of risk taking is the result of a strong loss aversion which leads to a preference for avoiding a sure loss, regardless of the probability of obtaining a gain while gambling. The powerful aversion to losses seems to have a stronger impact on risk perception than the incidental context manipulated by the presentation of fearful faces. Loss aversion leads to such a strong tendency to choose the risky option in the loss frame that the incidental fear cannot reduce risk taking. The absence of impact of the incidental anger condition could be explained by a ceiling effect of risky choices in the loss frame, as the percentage of risky choices observed in this study is at the same level as that observed in previous studies (De Martino et al., 2006; Talmi et al., 2010; Cheung and Mikels, 2011; Cassotti et al., 2012).

An alternative explanation for our findings might be found in the approach/avoidance framework. While fear has been associated with avoidance behaviors, anger is an approach motivated affect (Adams et al., 2006; Peterson and Harmon-Jones, 2012). Therefore, exposure to emotional context of fear might induce avoidance behaviors and then increase the likelihood of making risky decisions. In contrast, an emotional context of anger might induce approach behaviors and then decrease risky decisions. Although our results in the gain frame are in accordance with prediction of the approach/avoidance view, this model failed to fully explain the lack of emotional context effect in the loss frame. In addition, there is still a debate regarding whether fear triggers avoidance or approach behaviors (Hammer and Marsh, 2015).

Another possible explanation is that positive emotions have an impact on loss-aversion, whereas negative emotions have an influence on risk aversion. Previous studies have shown that positive emotions influence decision making in the loss frame (Cheung and Mikels, 2011; Cassotti et al., 2012). The preference for the gamble option in the loss frame is the result of loss-aversion that drives people to avoid sure losses. Thus, positive emotions may influence the disposition to loss aversion. Alternatively, this study seems to indicate that negative emotions influence decision making specifically in the gain frame. The preference for the sure option in the gain frame results from risk-aversion driving people to prefer a sure gain to a risky option of the same expected value. Thus, fear and anger seem to influence risk-aversion in opposite ways—risk-aversion increases in the incidental fear condition, whereas it decreases in the incidental anger condition—but do not influence loss-aversion. Heilman et al. (2010) have previously shown that naturally occurring negative emotions influence risk-aversion when confronted with a task of decision making under uncertainty, the Balloon Analog Risk Task. Further experiments are required to better understand which processes are involved in decision making when participants are confronted with an incidental negative context compared to an incidental positive context.

Together, these results reinforce the view that emotions play a crucial role in framing susceptibility (De Martino et al., 2006; Kahneman and Frederick, 2007) and extend previous results demonstrating that an incidental context could significantly influence susceptibility to framing (Cassotti et al., 2012). Furthermore, the current study provides evidence that different negative incidental emotional contexts (i.e., fear and anger) have opposite effects on risk-aversion tendencies. These results emphasize the importance of investigating the effect of specific negative emotions on decision making. That said, we do not yet have evidence that these contexts affect the participants’ ability to recognize the formal equivalence of the two frames (gain vs. loss). Thus, further investigations are required to determine whether these contexts influence participants’ emotional reactivity to gains and losses or their ability to overcome the framing effect. The measure of the skin conductance response and of the ability to express a differential autonomic response according to the frame could shed some light on the specific influences of the emotional context (De Martino et al., 2008). We could also use emotional and motivational scales to determine whether these results reflect changes in motivational drives, in appraisal tendencies, in the subjective value attributed to the options or in approach/avoidance tendencies.

Framing effects and people’s risk preferences vary as a function of task domains and according to the type of framing effect (Levin et al., 1998). Thus, it could be of great interest to examine whether the effects of incidental fear and anger evidenced in the current study can be replicated in other task domains (e.g., human lifesaving or personal money) or other framing effects (Levin et al., 1998).

A possible limitation of the present study concerns the absence of monetary incentives in the decisional task. However, the 20 catch trials allowed us to determine whether the participants were actively engaged in the task. Note that the participants who obtained a percentage of success lower than 85% on these catch trials were excluded from the final sample. In addition, the absence of real incentives is unlikely to explain the difference observed between the different incidental emotional conditions. Finally, studies offering no monetary incentives have reported the same pattern of results (see, e.g., Whitney et al., 2008; Reyna et al., 2011; Cassotti et al., 2012) as those offering monetary incentives (De Martino et al., 2006; Talmi et al., 2010; Cheung and Mikels, 2011).

## CONCLUSION

In summary, the current study is the first to provide empirical evidence for a role of negative incidental emotions on risk-aversion in the framing effect by showing that two incidental emotions of negative valence have opposite effects on risk seeking. Incidental fear increased risk-averse choices, whereas incidental anger increased risk-seeking and consequently led to a suppression of the framing effect. Notably, both negative emotions affected risk-aversion specifically in the gain frame. These results offer an experimental complement to the neuroimaging study by De Martino et al. (2006) and previous behavioral investigations (Cheung and Mikels, 2011; Cassotti et al., 2012) underlying the role of emotions in framing susceptibility.

### Conflict of Interest Statement

The authors declare that the research was conducted in the absence of any commercial or financial relationships that could be construed as a potential conflict of interest.
